# Battling Glioblastoma: A Novel Tyrosine Kinase Inhibitor with Multi-Dimensional Anti-Tumor Effect (Running Title: Cancer Cells Death Signalling Activation)

**DOI:** 10.3390/cells8121624

**Published:** 2019-12-12

**Authors:** Anisha Viswanathan, Aliyu Musa, Akshaya Murugesan, João R. Vale, Carlos A. M. Afonso, Saravanan Konda Mani, Olli Yli-Harja, Nuno R. Candeias, Meenakshisundaram Kandhavelu

**Affiliations:** 1 Molecular Signaling Lab, Faculty of Medicine and Health Technology, Tampere University, BioMeditech and Tays Cancer Center, Tampere University Hospital, P.O. Box 553, 33101 Tampere, Finland; anisha.viswanathan@tuni.fi (A.V.); akshaya.murugesan@tuni.fi (A.M.); 2 Predictive Medicine and Data Analytics Lab, Faculty of Medicine and Health Technology, Tampere University and BioMediTech, P.O. Box 553, 33101 Tampere, Finland; aliyu.musa@tuni.fi; 3 Department of Biotechnology, Lady Doak College, Madurai 625002, India; 4 Faculty of Engineering and Natural Sciences, Tampere University, 33101 Tampere, Finland; rafael.camposdovale@tuni.fi; 5 Instituto de Investigação do Medicamento (iMed.ULisboa), Faculdade de Farmácia, Universidade de Lisboa, Av. Prof. Gama Pinto, 1649-003 Lisboa, Portugal; carlosafonso@ff.ulisboa.pt; 6 Shenzhen Institutes of Advanced Technology, Chinese Academy of Sciences, Shenzhen 518055, China; saravananbioinform@gmail.com; 7 Computational Systems Biology Group, Faculty of Medicine and Health Technology, Tampere University and BioMediTech, P.O. Box 553, 33101 Tampere, Finland; olli.yli-harja@tuni.fi; 8 Institute for Systems Biology, 1441N 34th Street, Seattle, WA 98103-8904, USA

**Keywords:** glioblastoma, thioester, anti-angiogenesis, tyrosine kinase inhibitor

## Abstract

Glioblastoma (GB), a grade IV glioma, with high heterogeneity and chemoresistance, obligates a multidimensional antagonist to debilitate its competence. Considering the previous reports on thioesters as antitumor compounds, this paper investigates on use of this densely functionalized sulphur rich molecule as a potent anti-GB agent. Bio-evaluation of 12 novel compounds, containing α-thioether ketone and orthothioester functionalities, identified that five analogs exhibited better cytotoxic profile compared to standard drug cisplatin. Detailed toxicity studies of top compound were evaluated in two cell lines, using cell viability test, apoptotic activity, oxidative stress and caspase activation and RNA-sequencing analysis, to obtain a comprehensive molecular profile of drug activity. The most effective molecule presented half maximal inhibitory concentration (IC_50_) values of 27 μM and 23 μM against U87 and LN229 GB cells, respectively. Same compound effectively weakened various angiogenic pathways, mainly MAPK and JAK-STAT pathways, downregulating VEGF. Transcriptome analysis identified significant promotion of apoptotic genes, and genes involved in cell cycle arrest, with concurrent inhibition of various tyrosine kinase cascades and stress response genes. Docking and immunoblotting studies suggest EGFR as a strong target of the orthothioester identified. Therefore, orthothioesters can potentially serve as a multi-dimensional chemotherapeutic possessing strong cytotoxic, anti-angiogenic and chemo-sensitization activity, challenging glioblastoma pathogenesis.

## 1. Introduction

Glioblastoma (GB) is a type of brain tumor with almost 100% recurrence rate and high resistance to current treatment modalities. The high heterogeneity exhibited within GB tumors render it unresponsive to single-target cytotoxic/anti-angiogenic agents and demands a significant clinical need for new multi-dimensional oncogenic inhibitors for GB.

Angiogenesis is a significant feature of GB, attributed to the overexpression of vascular endothelial growth factor (*VEGF*). Few of the most significant proangiogenic regulators that stimulate angiogenesis indirectly by inducing *VEGF* mRNA expression include the growth factors epidermal growth factor (*EGF*), transforming growth factor (*TGF-α* and *TGF-β*), tumor necrosis factor α (*TNF-α*), keratinocyte growth factor, insulin-like growth factor I (*IGF-I*), fibroblast growth factor (*FGF*), platelet-derived growth factor/(*PDGF*), and cytokines (interleukin (*IL*)*-1α* and *IL-6*) and amplifications of *Ras*/*Raf* genes [1]. Multiple strategies have been developed to target VEGF/VEGF receptor (*VEGFR*)–mediated angiogenesis, including VEGF blockade, VEGF Trap, and suppression of VEGFR signaling via receptor tyrosine kinase inhibitors (TKIs) [2,3,4]. *EGFR* belongs to the *ErbB* family receptors of Class I receptor tyrosine kinases (*RTKs*). Almost 60% of glioblastoma patients have some kind of genomic alteration affecting EGFR pathway [5]. Downstream effects of *EGFR* is mediated through phosphoinositide 3-kinase (*PI3K*), mitogen-activated protein kinase (*MAPK*), signal transducer and activator of transcription 3 (*STAT3*) pathways, and *Src* family kinases [6]. A number of studies have focused on inhibiting both *VEGFR* and *EGFR* so as to improve drug efficiency, including monotherapy with a multi-targeted tyrosine kinase inhibitor (e.g., vandetanib, AEE788, BMS-690514) [7,8]. Other significant pathways regulating tumor angiogenesis directly or indirectly via VEGF includes MAPK pathway [9], JAK-STAT pathway [10,11], and PI3K-AKT [12] pathway. Thus, a multi-targeted chemoagent that can effectively sequester multiple pathways involved in VEGF regulation would be an effective solution to tackle tumor pathogenesis.

**Scheme 1:**
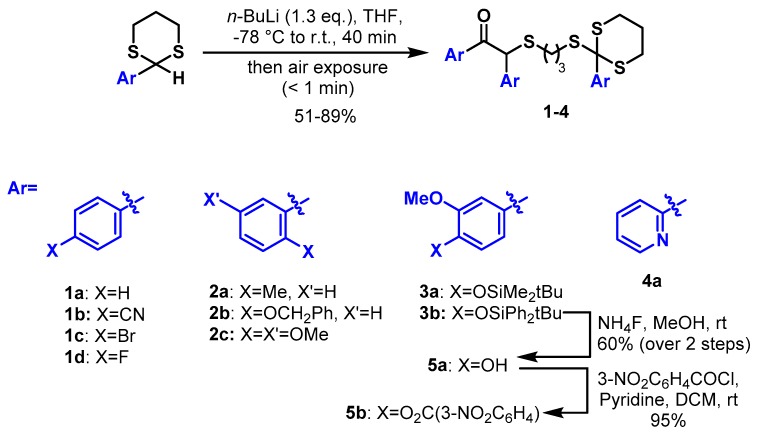
Synthesis and structures of studied orthothioesters.

Some of us have recently reported the unprecedented autoxidative condensation of 2-aryl-2-lithio-1,3-dithianes (Scheme 1) [13]. The result of such transformation was a small library of highly functionalized molecules containing α-thioether ketones and orthothioesters functionalities, among others. Motivated by the desire to find new agents capable of multi-target inhibition as promising approaches in the development of glioblastoma cancer drugs [14], we have set to assess the antitumor properties of these intricate molecules.

## 2. Materials and Methods

### 2.1. Synthesis of Orthothioesters

Reactions were monitored through thin-layer chromatography (TLC) with commercial silica gel plates (Merck silica gel, 60 F254). Visualization of the developed plates was performed under UV lights at 254 nm and by staining with cerium ammonium molybdate, 2,4-dinitrophenylhydrazine and vanillin stains. Flash column chromatography was performed on silica gel 60 (40–63 µm) as a stationary phase. NMR spectra were recorded with Varian Mercury 300 MHz or Jeol ECZR 500 instruments using CDCl_3_ as solvent and calibrated using tetramethylsilane as internal standard. Chemical shifts (δ) are reported in ppm referenced to the CDCl_3_ residual peak (δ 7.26) or TMS peak (δ 0.00) for ^1^H-NMR and to CDCl_3_ (δ 77.16) for ^13^C-NMR. The following abbreviations were used to describe peak splitting patterns: s = singlet, d = doublet, t = triplet, m = multiplet. Coupling constants, *J*, were reported in Hertz (Hz). High-resolution mass spectra (HR-MS) were recorded on a Waters ESI-TOF MS spectrometer. Tetrahydrofuran (THF) was dried by distillation under argon with sodium metal and benzophenone as indicator. Dichloromethane (DCM) was dried by distillation under argon with calcium hydride. Structural elucidation of all compounds tested in biological assays was performed by ^1^H and ^13^C-NMR, and HR-MS. All compounds of interest were isolated by chromatography and their purity (>97%) assessed by NMR.

Compounds **1**–**4** were prepared and characterized as reported elsewhere [13], through autoxidative condensation of 2-aryl-2-lithium-1,3-dithianes, prepared from treatment of 2-aryl-1,3-dithianes with *n*-BuLi followed by air exposure.

**5a**: (4-(1,3-Dithian-2-yl)-2-methoxyphenoxy)(*tert*-butyl)diphenylsilane (1.09 g, 2.27 mmol) was dissolved in dry THF (10 mL) in an argon purged round-bottom flask. The solution was cooled to −78 °C in an acetone/liquid nitrogen bath. *n*-BuLi (1.3 equivalents) was added dropwise to the reaction mixture at −78 °C. After addition, the solution was left stirring at −78 °C for 20 min, and then left to warm up to room temperature for 40 min. The argon balloon was replaced with an atmospheric air balloon and an additional needle was inserted in the septum as to allow air flow through the surface of the solution. After one minute, the solution was quenched with saturated aqueous NH_4_Cl solution (20 mL). Et_2_O (20 mL) was added and the layers separated. The organic phase was collected and the aqueous phase was extracted with Et_2_O (2 × 20 mL). The organic phases were combined and dried over MgSO_4_. The solvent was filtered and evaporated to yield crude 3b. The mixture was dissolved in dry methanol (8 mL) in an argon purged round-bottom flask. Then, ammonium fluoride (95 mg, 2.56 mmol, 1.1 equivalents) was added and the solution was stirred overnight at room temperature. The methanol was evaporated and water (10 mL) was added. The aqueous phase was extracted with DCM (3 × 10 mL) and the organic phases were combined and dried over MgSO_4_. The solvent was evaporated and the product was purified by flash chromatography, eluent hexane: ethyl acetate (1:1), to give product **5a** as an amorphous orange solid (286 mg, 0.45 mmol) in 60% yield. ^1^H-NMR (500 MHz, CDCl_3_) *δ* ppm 7.56–7.49 (m, 3H), 7.44 (dd, *J* = 8.4, 2.1 Hz, 1H), 6.98 (s, 1H), 6.89–6.82 (m, 4H), 6.07 (s, 1H), 5.68 (s, 1H), 5.61 (s, 1H), 5.45 (s, 1H), 3.90 (s, 3H), 3.89 (s, 3H), 3.86 (s, 3H), 3.27 (t, *J* = 12.5 Hz, 2H), 2.74–2.70 (m, 2H), 2.57–2.44 (m, 4H), 2.10–2.05 (m, 1H), 1.92–1.85 (m, 1H), 1.77–1.71 (m, 2H). ^13^C-NMR (125 MHz, CDCl_3_) *δ* ppm 194.0, 150.7, 147.1, 146.8, 146.4, 145.8, 145.6, 133.3, 128.8, 128.5, 124.3, 122.1, 121.3, 114.3, 114.0, 113.9, 111.0, 110.8, 110.6, 64.3, 56.2 (×2), 56.1, 55.2, 32.8, 30.7, 29.4, 28.5, 24.4. HR-MS (ESI) *m*/*z* calculated for C_30_H_34_O_7_S_4_Na^+^ [M + Na]^+^ 657.1080, found 657.1068.

**5b**: Triphenol **5a** (100 mg, 0.158 mmol) was dissolved in dry DCM (3 mL) in an argon purged round-bottom flask. Pyridine (48 µL, 0.591 mmol, 3.75 equivalents) was added to the solution, followed by 3-nitrobenzoyl chloride (91 mg, 0.488 mmol, 3.1 equivalents). The reaction was left stirring at room temperature for 72 h. Water (10 mL) was added to the mixture and the aqueous phase was extracted with DCM (3 × 10 mL). The organic phases were combined and dried over MgSO_4_. The solvent was evaporated and the product was purified by flash chromatography, eluent hexane: ethyl acetate (3:2), to give the benzoyl derivative **5b** as an amorphous white solid (163 mg, 0.151 mmol) in 95% yield. ^1^H NMR (500 MHz, CDCl_3_) *δ* ppm 9.02 (s, 3H), 8.51–8.48 (m, 6H), 7.76–7.67 (m, 6H), 7.62 (dd, *J* = 8.4, 2.0 Hz, 1H), 7.25–7.08 (m, 5H), 5.56 (s, 1H), 3.86 (s, 3H), 3.85 (s, 3H), 3.83 (s, 3H), 3.31 (dd, *J* = 13.3, 11.2 Hz, 2H), 2.79 (dd, *J* = 14.3, 3.6 Hz, 2H), 2.70–2.58 (m, 4H), 2.14–2.11 (m, 1H), 1.98–1.91 (m, 1H), 1.86–1.81 (m, 2H). ^13^C-NMR (125 MHz, CDCl_3_) *δ* 193.8, 162.6, 162.6, 162.2, 151.7, 151.6, 151.0, 148.5, 148.5, 143.8, 140.9, 139.5, 139.4, 136.1, 136.1, 135.9, 134.9, 131.2, 131.2, 130.8, 130.1, 130.0, 128.3, 128.1, 128.1, 125.5, 125.4, 122.9, 122.4, 122.3, 121.4, 120.6, 112.9, 112.8, 64.1, 56.3, 56.2, 56.2, 55.1, 32.8, 30.9, 29.5, 28.5, 24.3. HR-MS (ESI) *m*/*z* calculated for C_51_H_43_N_3_O_16_S_4_Na^+^ [M + Na]^+^ 1104.1418, found 1104.1385.

### 2.2. Cell Culture

Human GB cell lines, U87 cells were grown in Minimum Essential Medium (MEM, Product# 51416C, Sigma-Aldrich, St. Louis, MO, USA) with 10% Fetal Bovine Serum(FBS) 2 mM sodium pyruvate (Product# S8636, Sigma-Aldrich, St. Louis, MO), 1% Penicillin-Streptomycin and 0.025 mg/mL Amphotericin B. LN229 and non-cancerous cell line, mouse embryonic fibroblast (MEF) cells were cultured in Dulbecco’s Modified Eagle Medium—high glucose (DMEM, Catalog# L0102, Biowest, Riverside, CA, USA) containing 5% FBS (Product # F1051, Sigma-Aldrich, St. Louis, MO), 1% Penicillin-Streptomycin (Product # P4333, Sigma-Aldrich, St. Louis, MO) and 0.025 mg/mL Amphotericin B (Sigma-Aldrich, St. Louis, MO). U87 and LN229 are the standard cell lines derived from malignant gliomas used commonly for cytotoxicity study. MEF Cells were maintained at 37 °C in a humidified incubator supplemented with 5% CO_2_. Three biological and technical repeats were used for each condition.

### 2.3. In Vitro Cytotoxicity Assay

Cytotoxicity assay was performed to determine the cell growth inhibitory effect of the compounds following treatment for 48 h on the GB cell lines, U87, LN229. This assay was performed in two stages. At first, a high concentration, 100 µM, of compounds were used as well as for the positive control Cisplatin, CIS (Sigma-Aldrich, USA). Following this, the top compound was selected, and different concentrations (100 µM, 75 µM, 50 µM, 25 µM, and 10 µM) of the compound were tested to determine the IC_50_. Treated cells were harvested by centrifugation at 1200 rpm for 10 min. Cell viability was determined using trypan blue staining. The number of live and dead cells were counted using a Countess II Automated Cell Counter (Thermo Fisher Scientific, Carlsbad, CA, USA). The proliferation inhibition percentage of each sample was determined using the following formula to determine the dose-response curve. From the dose-response curve, the IC_50_ value of each compound was calculated. The cytotoxicity of the top compound and Cisplatin at a concentration of 10 µM was also evaluated in normal brain cells (MEF). Percentage of inhibition of cell proliferation was calculated using the following formula:

$$\begin{array}{c} Proliferation\;inhibition\;\left(\%\right)\\ =\frac{Mean\;No.\;of\;untreated\;cells\;\left(DMSO\;control\right)-Mean\;No.\;of\;treated\;cells\;\times 100}{Mean\;No.\;of\;untreated\;cells\;\left(DMSO\;control\right)} \end{array}$$

### 2.4. Double Staining Assay

U87 and LN229 cell lines were grown as described previously, followed by cells treatment with IC_50_ concentration of 5a and incubated for 48 h. Untreated (Negative) and Cisplatin (positive) controls were also maintained. Apoptosis/necrosis detection was carried out using Annexin V-FITC and PI (Thermo Fisher Scientific). The apoptosis determination was performed following the standard protocol suggest by the manufacturer. Briefly, the cells were cultured in 6-well plate with an initial cell density of 7 × 10^5^ cells/well. The cells were incubated for 48 h with 5a, positive control and untreated cells conditions and then harvested and washed in cold phosphate buffered saline (PBS). The cell pellets were then resuspended in 1× Annexin-binding buffer provided in the kit. Consequently, 5 μL of FITC conjugated Annexin V and 1 μL of the 100 μg/mL PI working solutions were added to the 100 μL of cell suspension. The cells were incubated in dark for 15 min, at room temperature, after which the stained cells were observed for fluorescence to distinguish apoptotic cells. The image acquisition was done by using an EVOS imaging system (ThermoFisher Scientific) with 20× objective magnification. More than 300 cells were used for each analysis. The percentage of apoptosis was quantified based on the cells stained with Annexin V-FITC positive and PI negative and both Annexin V-FITC and PI positive. The percentage of necrosis was calculated based on the cells with Annexin V-FITC negative and PI positive [15,16,17]. The fold change in apoptosis was calculated against the untreated cells.

### 2.5. Caspase Activity Assay

In-vitro caspase-3 and caspase-7 activity was determined using Caspase-Glo^®^ 3/7 Assay Systems (Promega Corporation, Madison, WI, USA). The reagent was prepared as mentioned in the manufacturer protocol. The U87 and LN229 cells were grown overnight in a 96-well plate and were treated with an IC_50_ of 5A. Negative control, positive control and blank (medium+ Dimethyl sulfoxide (DMSO)+ dye) were also maintained. Cells were incubated at 37 °C in a humidified incubator supplemented with 5% CO_2_ for 5 h and then equilibrated at room temperature for 30 min. 100 µL of Caspase-Glo 3/7 reagent was added to 100 µL of cells/well and was incubated in a dark-chamber. The luminescence signal was quantified (Chameleon Multi-label Detection Platform) at one hour after treatment. Magnitude of fold change in luminescence between treated and untreated cells were determined using the following formula:

$$Fold\;increase=\frac{F_{test}-F_{blank}}{F_{control}-F_{blank}}$$

### 2.6. Intracellular Redox Potential Test

To evaluate the redox potential of 5a, a comparative test, using H_2_O_2_ and standard drug against untreated cells, was performed using H2DCFDA (Catalog no.# D399 Life Technologies, Eugene, OR, USA). The U87 and LN229 cells were grown overnight in a 96-well plate and were treated with an IC_50_ of 5a for 5 h at 37 °C in a humidified incubator supplemented with 5% CO_2_. Negative control, positive control and blank were maintained. Baseline effect due to solvent was determined as well. After 5 h of treatment, cells were harvested by centrifugation at 3000 rpm for 10 min and incubated with 200 µL of 2 µM H2DCFDA for 30 min at 37 °C in the CO_2_ incubator. Cells were then washed with pre-warmed PBS and resuspended in 200 µL of pre-warmed medium. Next, 100 µL of suspension was transferred to each well, in a 96-well plate and incubated at room temperature for 20 min. Finally, fluorescence signal was measured using Chameleon Multi-label Detection Platform (Excitation 485 nm, Emission 535 nm).

### 2.7. RNA Isolation and Gene Expression Evaluation

Cell were incubated with IC_50_ of test compound and standard drug Cisplatin for 48 h at 37 °C in a humidified incubator supplemented with 5% CO_2._ A negative control was maintained as well. All conditions were conducted in triplicated samples for the isolation of RNA. Total RNA of >9.25 ng/µL was isolated using GeneJET RNA purification kit (Catalog no #K0731), according to the manufacturer’s instructions. The yield was then measured spectrophotometrically using NanoDrop-1000 (Thermoscientific, Wilmington, NC, USA). After quantification, the cells were considered for quality assessment by TapeStation. The gene expression analysis was performed by using Illumina Next Seq High throughput profiling (Illumina NextSeq 500). The sequencing produced data in bcl format which was converted into FASTQ file format.

### 2.8. Transcriptome Analysis

Differential expression (DE) and statistical analysis were performed using DESeq2 [18] (release 3.3) in R (version 3.2.4) (https://bioconductor.org/packages/release/bioc/vignettes/DESeq2/inst/doc/DESeq2.html). *p*-values were adjusted for multiple testing using the Benjamini-Hochberg procedure [19]. A false discovery rate adjusted *p*-value (i.e., *q*-value) < 0.05 was set for the selection of DE genes.

### 2.9. Phosphorylation of MAPKs and Other Serine/Threonine Kinases

The U87 cells were treated with an IC_50_ concentration of **5a** and DMSO, maintained in CO_2_ humidified temperature for 48 h. The total protein was extracted using RnD protein extraction kit for the immunoblotting experiment. The phosphorylation of three families of mitogen-activated protein kinases (*MAPKs*), including the extracellular signal-regulated kinases (*ERK1/2*), c-Jun N-terminal kinases (*JNK1-3*), and different p38 isoforms, was analyzed following the manufacturer protocol, RnD Human Phospho-MAPK array kit. In detail, cell lysates are mixed with a cocktail of biotinylated detection antibodies and incubated with Proteome Profiler Human Phospho-MAPK Array. Streptavidin-HRP and chemiluminescent detection reagents are added and the signals that are produced at each spot correspond to the amount of phosphorylated protein bound. The captured and control antibodies have been spotted in duplicate on nitrocellulose membranes. The signals are measured using ChemiPro Luminescence detection system. The relative level of phosphorylation was analysed for 26 *MAPK* proteins as described in the manufacturer’s protocol.

### 2.10. Gene Ontology (GO) and Pathway Analysis

Gene ontology [20] and ClusterProfiler package [21] was used for pathway analyses. We performed with the over-representation test for the GO biological process and KEGG pathways [22]. The package supports the human genome. We used the binomial test and Bonferroni correction for multiple testing and displayed z-scores to indicate whether a potential regulator was activated or inhibited. We used the default settings for statistical analysis in both the KEGG pathways and GO terms. In this analysis, pathways and GO terms only with a *p*-value < 0.05 were included.

### 2.11. Evaluation of Structure-Activity Relationship

In order to make a comparative study on the binding of **5a** with different receptors, sequences and structures of six receptors taken from Protein Data Bank (PDB)—Fibroblast Growth Factor receptor (*FGFR*, PDB ID: 2FDB), Epidermal growth Factor Receptor (*EGFR*, PDB ID: 4UIP), Platelet-Derived Growth Factor Receptor (*PDGFR*, PDB ID: 1PDG), *c-MET* Receptor (PDB ID: 3DKC), c-KIT Receptors (PDB ID: 6GQK) and Vascular Endothelial Growth Factor receptors (PDB ID: 3V2A) were obtained from the Protein Data bank. To study the binding efficiency and to identify the important amino acid residues contribute to the binding, Patchdock molecular docking program was used. The default parameters were used for the molecular docking in this study. Patchdock carries out rigid docking, with surface variability implicitly addressed through liberal intermolecular penetration.

### 2.12. Statistical Analysis

All experiments described in the present study were performed as with three biological and technical repeats. The data were presented as the mean ± standard error of mean. Statistical analyses between two groups were performed by Student’s *t*-test. Differences among multiple groups were tested by one-way analysis of variance following by a Dunnett’s multiple comparison test (GraphPad Prism 7.04, San Diego, CA, USA). *p* < 0.05 was considered to indicate a statistically significant difference.

## 3. Results

### 3.1. Synthesis of Orthothioesters

The orthothioesters **1**-**4** were synthesized through autoxidative condensation of 2-aryl-2-lithium-1,3-dithianes, prepared in situ from treatment of 2-aryl-1,3-dithianes with *n*-BuLi followed by air exposure. The presence of bromide when preparing **1c** demanded for replacement of *n*-BuLi by lithium diisopropylamide (LDA) in order to avoid transmetalation. The compounds were obtained in reasonable yields and pure after quenching with NH_4_Cl saturated aqueous solution, usual work-up and purification by silica chromatography. The silyl ether group in compound **3b** was cleaved by treatment with 1 equivalent of NH_4_F to yield phenol **5a**. Derivatization of **5a** with *m*-nitrobenzoyl chloride delivered carboxylic ester **5b** in excellent yield. Purity evaluation results of the novel compounds are given in the Supplementary file 1.

### 3.2. **5a** Inhibited Proliferation of Glioma Cells

**Figure 1:**
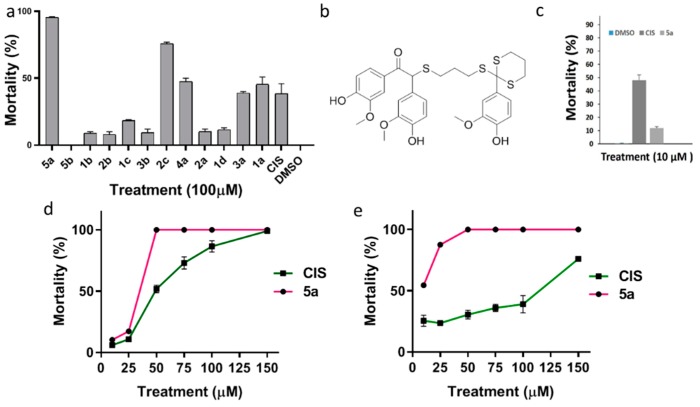
In vitro screening and evaluation of dose response of thioester compounds (**a**). The percentage of mortality rate on treating U87 cells with novel panel of 12 thioester derivatives along with CIS (positive control) 1% DMSO (negative control), at a 100 µM concentration (**b**). Chemical structure of the investigative drug **5a**. (**c**) Cytotoxicity analysis of non-cancerous brain cells, MEF and cisplatin at 10 µM concentration. (**d**) Dose response curve of U87 cells (left) and (**e**) LN229 (right) cells treated with the top compound (**5a**), CIS and 1% DMSO was analyzed to determine IC_50_ value. Treatment was carried out at 48h and cellular viability was measured by Trypan Blue exclusion method. Data points and error bars represent mean ± standard error of the mean (SEM) (n = 6 per group). Significant when *p* < 0.05, one-way ANOVA.

The cytotoxicity of the orthothioesters **1**-**5** against the growth of U87 GB cell line was performed with 100 μM concentration of the compounds as described in the method section. Upon evaluation of the 12 compounds depicted in Scheme 1, **5a** was identified to be the most cytotoxic compound inducing 90% cell death and **5b** was the least cytotoxic with 0% cell death. This was a 20-fold higher activity compared to the standard drug cisplatin and 31.8-fold compared to the negative control (Figure 1a) The analysis of cytotoxic data of all compounds tested shows that while the presence of halogens or nitrile in the 4-position of the aromatic rings is detrimental for cytotoxicity (as for compounds **1b**–**d**), having a methoxy group in the 3-position has an opposite effect. Compounds **2c**, **3a** and **5a**, decorated with a 3-methoxy substituent in the aromatic rings, showed cytotoxicity similar to or higher than **1a**. Compounds **3b** and **5b** on the other hand, also decorated with the same 3-methoxy substituent but having bulky substituents in the vicinal 4-position, have residual or absent cytotoxicity. No improvement on cytotoxicity was observed when replacing the aryl groups by heteroaryl such as pyridyl in **4a**. The cytotoxicity on normal brain cells, MEF, was also performed for the top compound **5a** (Figure 1b) and cisplatin at a concentration of 10 μM. The results show that **5a** have 11% of growth inhibition, whereas cisplatin have 48% of inhibition, suggesting that **5a** is more toxic to the cancer cells than the normal cells (Figure 1c).

In order to investigate the in vitro anticancer activity of **5a** and to determine its IC_50_ value, we evaluated cytotoxicity against cell lines, U87 and LN229 in the presence of increasing concentrations of this compound for 24 h, ranging from 0 μM–150 μM. The viability of cells was determined according to cell counting based on Trypan Blue assay and the IC_50_ value of **5a** was determined as 27 μM in U87 cells and 23 μM in LN229 cells (Figure 1d,e). The IC_50_ value of cisplatin was identified as 53 μM in U87 cells and 115 μM in LN229.

### 3.3. **5a** Induced Negligible Oxidative Stress and Promoted Caspase Activation

**Figure 2:**
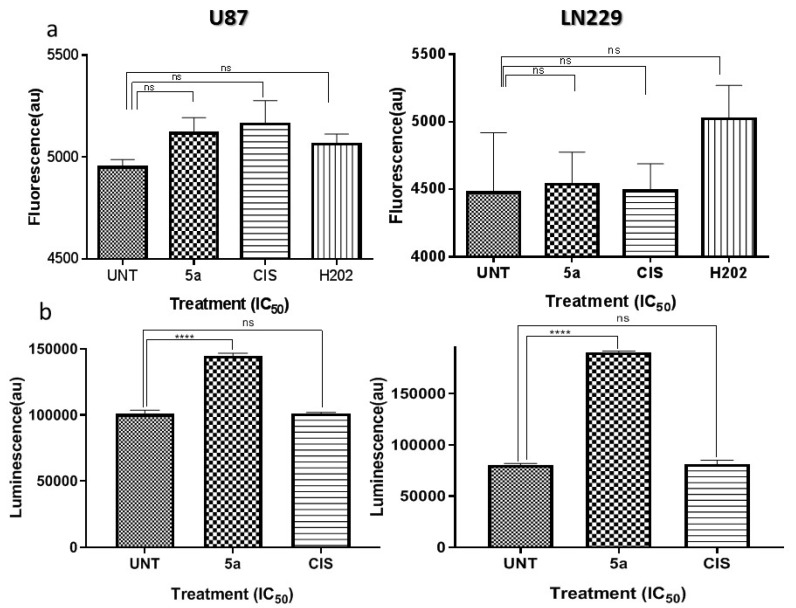
Comparative evaluation of oxidative stress response and caspase activation by the **5a** in two different cell lines (**a**) Effect of **5a** on intracellular reactive oxygen species (ROS) production by GB cells as interpreted by fluorescence level. Evaluation suggested no significant increase in ROS level upon drug treatment (α = 0.05). (**b**) Caspase activity displayed by GB cells analyzed using caspase 3/7 luminescence assay. Significant increase in caspase activity of cells was observed after treatment with IC_50_ of **5a** for 5h compared to control. The values are expressed as means ± standard error of the mean (SEM) of triplicate measurements of biological repeats (n = 3). Significance: **** *p* < 0.0001.

Reactive oxygen species (ROS) mediated caspase activation of tumor cells during stress and subsequent cell death has been repeatedly reported by various studies [23,24]. In the present study, both intracellular ROS and caspase in U87 cells were quantified to verify oxidative stress and cellular response upon **5a** treatment. After exposure of U87 cells to **5a** at IC_50_ for 5 h, we detected an oxidative increase of 3.3% increase in the **5a** treated cells when compared to untreated cells. The standard drug cisplatin and positive control H_2_O_2_ on the other hand, marked a 4.2% and 2.2% increase in oxidative response respectively (Figure 2a). Similarly, exposure of LN229 cells to **5a** at IC_50_ for 5 h demonstrated negligible change in the treated cells when compared to untreated cells (Figure 2a). The difference between treated and untreated conditions was confirmed to be statistically insignificant per ANOVA test (*p*-value <0.05), establishing that the **5a** does not increase ROS in the tested GB cells.

Considering the role of Caspase activation, we determined the caspase activity of U87 cells using caspase3/7 assay after a treatment period of 5 h with **5a** at IC_50_. Interestingly, U87 **5a**-treated cells displayed an increase of caspase 3/7, displaying a 0.43-fold increase in comparison to untreated cancer cells, whereas positive control displayed no significant increase (0.003) in caspase activity (Figure 2b). Similarly, in LN229 cells **5a**-treated cells demonstrated 1.37-fold increase with respect to untreated cells, whereas the standard drug cisplatin exhibited 0.05 fold increase in caspase levels. The difference between treated and untreated conditions was confirmed to be statistically significant per ANOVA test (*p*-value < 0.05).

### 3.4. **5a** Enhanced Apoptosis and Cell Cycle Arrest

Defective apoptotic machinery enhances tumor pathogenesis by permitting survival of genetically unstable cells leading to treatment resistance. To understand if the decrease in viability was due to apoptosis, we treated U87 cells with IC_50_ of 5a and we determined the apoptosis effect using double staining method.

**Figure 3:**
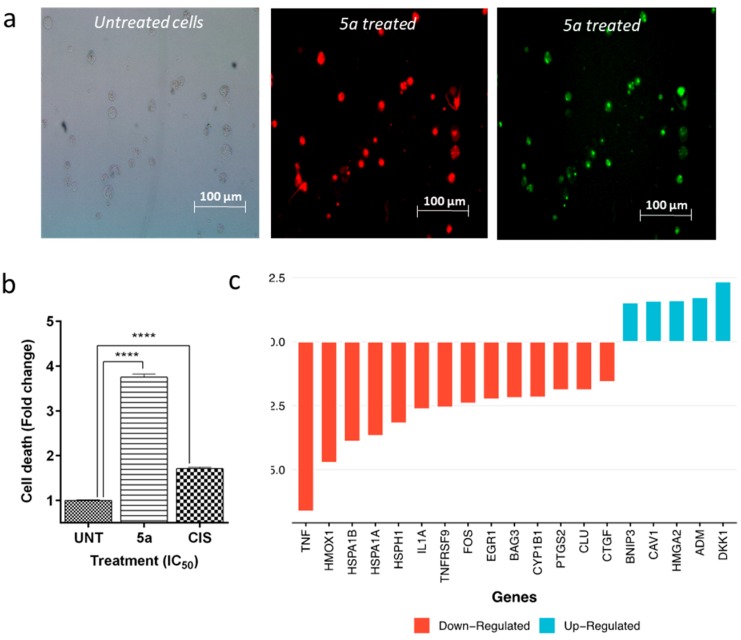
Evaluation of apoptotic activity induced by **5a**. (**a**). Phase contrast image of non-apoptotic cells in untreated condition (left); fluorescence image of U87 cells exhibited upon PI (middle) and Annexin V (right) staining. **5a** was found to effectively induce apoptotic cell death when compared to untreated cells. (**b**) The histogram represents quantification of apoptosis after treating the cell lines with IC_50_ of **5a**, for 48 h, in U87 cell line. Cisplatin was used as a positive control and untreated cells as negative control. The fold change of apoptosis has been calculated against untreated U87 cells. The values are expressed as means ± standard error of the mean (SEM) of triplicate measurements of biological repeats (n = 3). **** *p* < 0.0001 as produced by ANOVA test. (**c**) Key genes involved in Apoptotic process and their log2(fold-change), that are differentially expressed upon R114 treatment, compared to untreated. log 2(fold-change) cutoff = 1.5.

**Figure 4:**
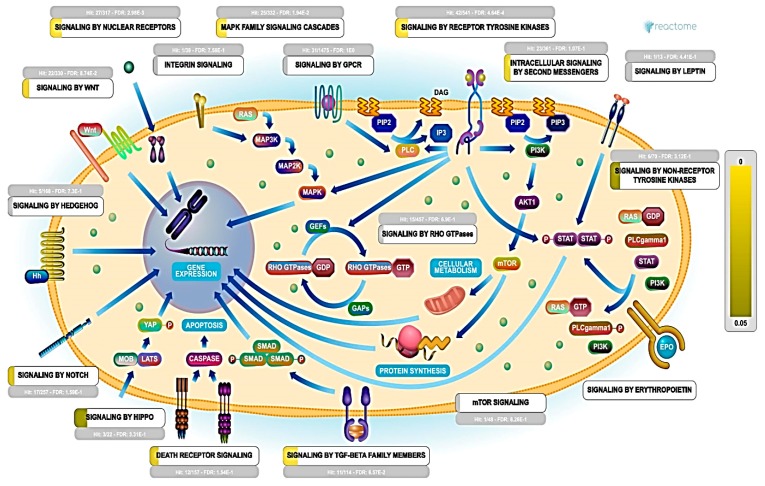
Representation of DEGs in major signal transduction pathways, in **5a** treated cells. The color code denotes over-representation of that pathway by DEGs. FDR represents the corrected over-representation probability. Color scale denotes the proportion of entities identified among the total entities enriched in a pathway (Image generated via the reactome pathway analysis tool; https://reactome.org/PathwayBrowser/).

**Figure 5:**
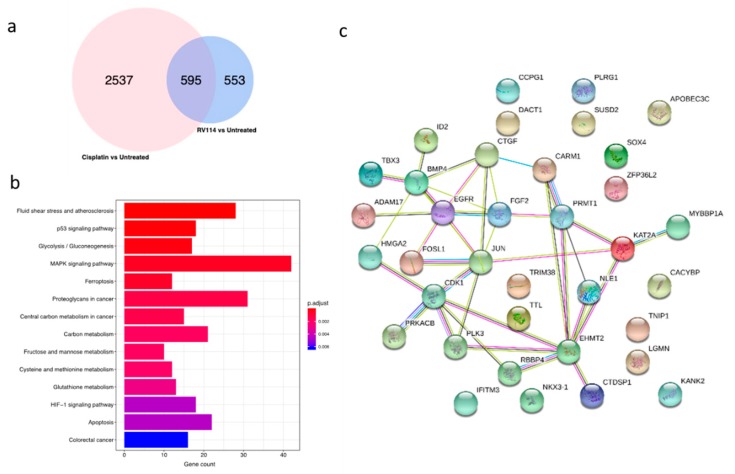
Transcriptional profile of **5a** compared to standard drug (**a**) Venn diagram showing 595 genes commonly differentially expressed in both treatments versus control, as assessed by RNA-seq data analysis of U87 cell line. (**b**) Visual representation of top 14 pathways associated with differentially expressed genes upon **5a** treatment with reference to control. (**c**) Gene interaction network of cell cycle genes affected upon **5a** treatment.

The results revealed that **5a** was much effective in inducing apoptosis exhibiting a 3.75-fold increase in programmed cell death, whereas the standard drug cisplatin induced a 1.7-fold increase when compared to untreated cells (Figure 3a,b). To explore in detail the effect of 5a transcriptomics, data analysis was carried out, and we identified 1148 differentially expressed genes (DEGs) when **5a** is compared with untreated (negative control) samples (*q*-value < 0.05). We also compared the **5a** and cisplatin samples as a positive control group, both individually and combined as a single “affected” group. In these comparisons, 3132 DEGs were identified, with the largest number of DEGs identified in comparison with the cisplatin samples. In total, 595 out of 4280 DEGs were common in both comparisons. The complete lists of DEGs from the cell line analysis and all pairs of comparisons are provided in Supplementary file 2. Gene expression analysis indicated that **5a** has a multidimensional impact on various tumorogenic features, especially on tyrosine kinase signaling (Figure 4 and Figure 5a) and was effective in increasing expression of agonists of cell death such as *DKK1*, *ADM*, *HMGA2*, and *CAV1*. In addition, downregulation of chemoprotective factors such as *HMOX1*, *HSPs*, and *CYP1B1* were detected. Adding to its cytotoxic effects, **5a** was also found to suppress mitochondrial membrane stability as evident by dysregulated levels of *HSPA1A* and *BNIP3*.

Gene expression analysis also points out that treatment of U87 cells with **5a** leads to inhibition of G1/S transition leading to cell cycle arrest. Upregulation of cell cycle inhibitory genes involved in G1/S such as *DACT1*, *SUSD2*, and *CTDSP1* signifies the efficiency of the drug as a cell cycle inhibitor (Figure 5c). Additionally, genes favoring G1/S transition such as *PLRG1* and *ADAM17* were effectively downregulated due to **5a** treatment. The cell cycle interruption is further strengthened by suppression of *CCND3* affecting the *CDK4* activity associated with this cyclin, which is necessary for cell cycle progression. Several studies have proposed that agents that interfere with DNA repair can act as a therapeutic strategy targeting double-strand break repair pathways or abrogate cell cycle checkpoints. *HMGA2* gene involved in negative regulation of double-strand break repair via non-homologous end joining was found to be risen upon the treatment with **5a** demonstrating its genotoxicity leading to the apoptotic death of cancer cells. Additionally, notable chemo-sensitizing was visible in treated cells as evident from transcriptome levels of *HSPA1*, *CLU*, and *TXN* (Table 1).

### 3.5. **5a** Is an Anti-Angiogenic Agent

To gain more insight on anti-angiogenic efficacy of **5a**, we analyzed the modulation of genes in U87 glioma cells treated with the investigative drug. **5a** was found to adversely affect the cytokine receptor pathway exerting its effect through interaction of multiple pathways, eventually downregulating *VEGF* expression. *VEGFD* itself exhibited a log_2_ (fold change) of −4.5. Other key genes (*TNF*, *HMOX*, *IL1A*, *CYP1B1*, and *PTGS1*) involved in positive regulation of *VEGF* were identified to be downregulated by more than 1.75-fold. Key genes involved in inhibition of angiogenesis, including *NR2F1*, *SEM3A*, *ERRFI1*, *SPRY1*, *ADM*, *NRP1*, etc., were found to be upregulated (log_2_ (fold change) > 1.5). Moreover, enhancers of migration and angiogenesis such as *GPNMB*, *PTGS2*, *CD274*, and *ZNF703* were significantly downregulated suggesting suppression of tumor malignancy (Table 1 and Supplementary file 2).

**Figure 6:**
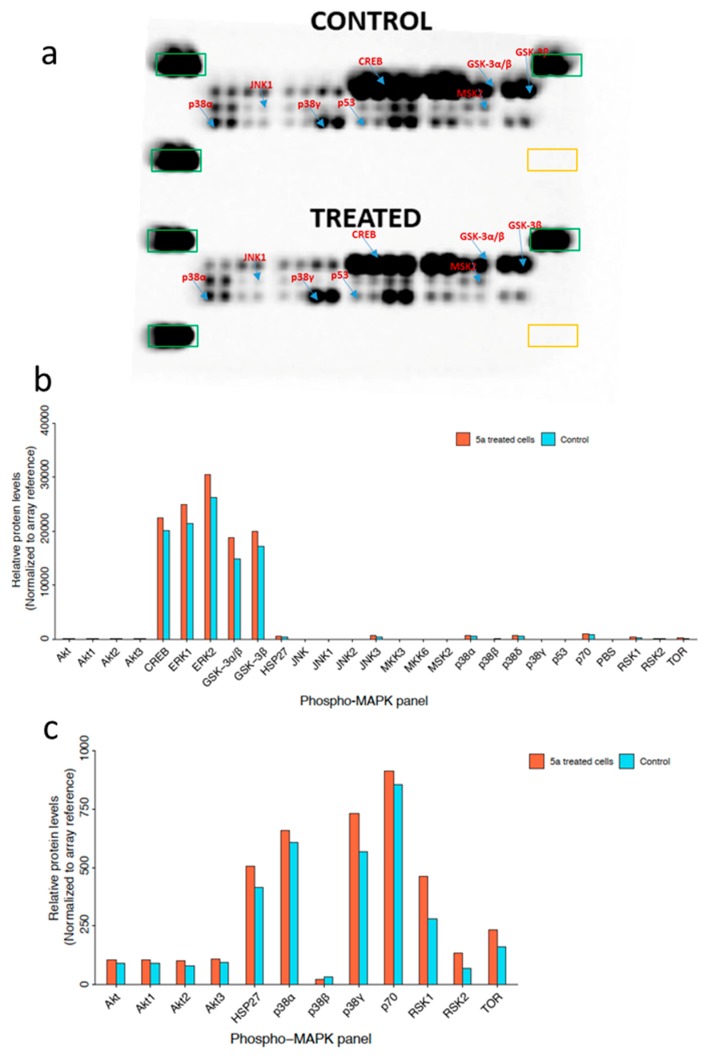
Analysis of phosphor–MAPK array. (**a**) The array images are shown for both DMSO control and **5a** treated cells and the eight phosphorylated proteins are marked in blue arrow. Positive reference spots are represented in green box and the negative reference spot (PBS) is represented in yellow box. (**b**) The complete set of *MAPKs* was presented according to the relative level of intensity of phosphorylation. (**c**) Absolute values of integrated pixel intensity of a few kinases are displayed for better resolution.

**5a** advocates inhibition of angiogenesis targeting VEGF pathway, via multiple pro-angiogenic regulators. The mRNA levels of *VEGF* were markedly decreased in the U87 cells treated with the IC_50_ concentrations of **5a**. Moreover, other angiogenic enhancers such as *HMOX1*, *IL1A*, *CYP1B1 PGF*, and *FGF* were effectively suppressed by **5a**. Additionally, such a molecule potentially weakened angiogenesis by affecting pathways involved in the crosstalk by interrupting key regulators involved. The phosphorylation of multiple kinase proteins involved in the VEGF signaling pathway were quantified using immunoblotting. The upregulation of *CREB*, *GSK-3α/β*, *GSK-3β*, *MSK2*, *p38α*, *p38γ*, and *p53* and down regulation of *JNK1* validates that the VEGF pathway might be targeted by **5a** (Figure 6a–c).

### 3.6. **5a** Restricts MAPK Signaling Cascade and Downregulates TNF Expression

Activation of the MAPK pathway leads to the transcription of genes that encode proteins involved in the regulation of essential cellular functions, such as cell growth, cell proliferation, and cell differentiation [25]. U87 glioma cells treated with IC_50_ concentrations of **5a** exhibited downregulation of positive regulators of MAPK pathway such as *GADDs*, *RIPK*, *CTNNB1* and *PSAP*, whereas, it enhanced the expression of inhibitors of MAPK signaling such as *MYC*, *SPRY1*, *EZR*, *RASA3* and *CAV1*. Additionally, **5a** reduced the expression of activators of ERK cascade (*TNF*, *CTGF*, *FGF*, *JUN*) and enhanced its negative modulators such as *C1QL4* and *TNIP1* (Figure 5b, Table 1 and Supplementary file 2). *TNF* exerts its biological functions by activating distinct signaling pathways such as nuclear factor κB (*NF-κB*) and c-Jun N-terminal kinase (*JNK*). We observed a significant reduction of TNF levels in 5a treated cells compared to untreated cells. Inhibitors of TNF production like *ERRIF1*, *RARA*, *VSIR* and *AXL* were found to be overexpressed explaining the reduction in TNF level. Additionally, immunoblotting revealed the phosphorylation of *CREB*, *p38*, *JNK* proteins involved in MAPK/TNF signaling pathway. The relative higher-level expression of *CREB*, *p38* and reduced expression of *JNK* protein, also validates the inhibition of MAPK signaling cascade by **5a** in U87 cells (Figure 6a–c).

### 3.7. **5a** Negatively Affects JAK-STAT Pathway

The direct and mediated mechanisms of JAK-STAT signaling in tumor cell survival, proliferation, and invasion have made the JAK-STAT pathway a feasible target for drug development and cancer therapy. Interactions of JAK/STAT pathway with the RTK/Ras/MAPK pathway, TGF- β signaling pathway and PI3K pathway amplifies the effect mediated through the regulation of JAK-STAT signaling [26]. The current study revealed that **5a** is a strong suppressor of JAK-STAT pathway causing notable downregulation of major and downstream genes such as *STAT1*, *MCL1*, *CCND3*, *CDKN1A*, and *IL15* (Table 1 and Supplementary file 2). Upregulation of *PTPN2*, an inhibitor of *JAK* expression was observed, which is also a contributor of endoplasmic reticulum stress-induced intrinsic apoptotic signaling pathway. Additionally, upregulation of *CAV1* and *HMGA* was identified explaining the downregulation of JAK-STAT pathway. Phosphorylation analysis of multiple kinases shows the upregulation of *CREB* and *p38* proteins, suggesting a role of **5a** in inhibiting JAK-STAT signaling pathway (Figure 6a–c).

**Figure 7:**
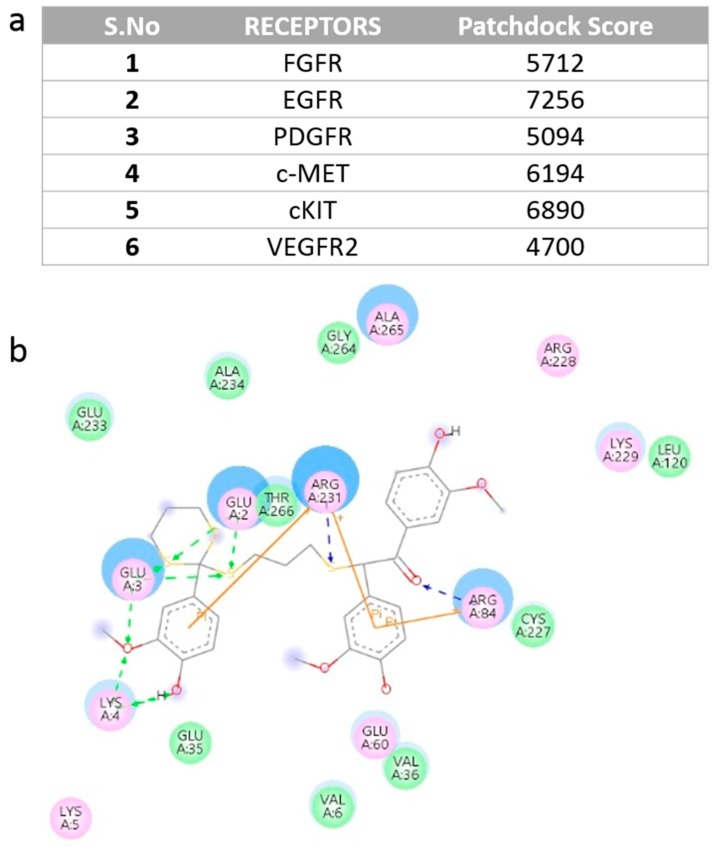
Docking score of **5a** with various kinase receptors (**a**) Docking analysis of various cytokine receptors (Fibroblast Growth Factor receptor—*FGFR*, PDB ID: 2FDB, Epidermal growth Factor Receptor—*EGFR*, PDB ID: 4UIP, Platelet-Derived Growth Factor Receptor—*PDGFR*, PDB ID: 1PDG, c-MET Receptor, PDB ID: 3DKC, *c-KIT* Receptor- PDB ID: 6GQK, and Vascular Endothelial Growth Factor receptors PDB ID: 3V2A) with **5a**. Docking study was carried out using the Patchdock program. (**b**) 2-dimensional interaction diagram of EGFR with ligand.

Several cross-family interactions among tyrosine kinases may significantly alter angiogenic signaling, leading to anti-angiogenic drug resistance. Reports say *FGF*-driven angiogenesis is blocked by *VEGF* inhibition, which suggests that *FGF* controls angiogenesis upstream of *VEGF* by modulating *VEGF* function [27]. Additionally, in the case of glioma tumorigenesis, *PDGF*- expression is assumed to contribute to the expansion of an established tumor as well as the regulation of the angiogenic switch for initial tumor development [28]. Studies also testify that there are cross family interactions between *VEGF* and *PDGFR* [29]. In addition, *cKIT* and *MET* are another two important *RTKs* to be explored as angiogenic drivers [30]. c-KIT signaling promotes cell proliferation and survival, exerting its effect through Ras-Erk pathway as well as JAK/STAT pathway [31]. Chen et al. asserted that intracrine *VEGF* function can be regulated by MET signaling and plays a significant role in controlling *VEGFR2* [32]. In this context, we sought to explore interactions between promising orthothioester 5a and six different tyrosine kinase receptors-*FGFR*, *EGFR*, *PDGFR*, *c-MET*, *cKIT*, and *VEGFR-2*, by in silico docking study. The results show that the Epidermal growth Factor Receptor shows good binding efficiency with thioester with high docking score (7256) (Figure 7a). The two-dimensional ligand interaction diagram of thioester with *EGFR* is shown in Figure 7b. Conclusively, our study evidences a multidimensional anti-tumor effect of the novel thioester drug **5a**.

### 3.8. **5a** Induced Phosphorylation of MAPKs and Other Serine/Threonine Kinases

The effect of **5a** on the phosphorylation levels of three families of *MAPKs*, such as *ERK1/2*, *JNK1–3* and different p38 isoforms, was analyzed. The arrays used in the experiments are based on the analysis of kinase specific antibodies which are spotted in duplicates along with three reference spots and PBS as negative spots. The mixture of biotinylated anti-phospho-kinase antibodies and streptavidin-HRP conjugate differentiates the phosphorylated and non-phosphorylated protein between the control and **5a** treated cells. The luminescence induced by the addition of chemiluminescence reagent was captured using the ChemiPro detection system (Figure 6a). In detail, the relative levels of phosphorylation of 26 kinases such as *Akt1*, *Akt2*, *Akt3*, *Akt*, *CREB*, *ERK1*, *ERK2*, *GSK-3α/β*, *GSK-3β*, *HSP27*, *JNK1*, *JNK2*, *JNK3*, *JNK*, *MKK3*, *MKK6*, *MSK2*, *p38α*, *p38β*, *p38δ*, *p38γ*, *p53*, *p70*, *RSK1*, *RSK2*, and *TOR* (Figure 7b) were analyzed. The phosphorylation level of *CREB* (Ser^133^), *GSK-3α/β* (Ser^21^/Ser^9^), *GSK-3β* (Ser^9^), *MSK2* (Ser^360^), *p38α* (Thr^180^/Tyr^182^), *p38γ* (Thr^183^/Tyr^185^) and *p53* (Ser^46^) were significantly increased in 5a treated U87 cells, whereas the phosphorylation level for *JNK1* (Thr^183^/Tyr^185^) was found to be reduced relative to the control (Figure 6c). The results suggest that the EGFR signaling pathway was suppressed by **5a**, which is confirmed by the phosphorylation of not only the *MAPKs* and other serine threonine kinases. Thus, the **5a** suppression of *EGFR* further substantiates that 5a as TK inhibitor.

## 4. Discussion

In order to challenge tumor recurrence and resistance of GB, agents that can synergistically confront multiple oncogenic pathways are gaining great importance in GB drug discovery. In a pursuit to identify a multi-targeted chemo-agent against glioblastoma, we assessed bioactivity of a panel of new orthothioester derivatives as anti-GB agents in vitro.

Cytotoxicity evaluation identified an orthothioester **5a**, which has inhibited tumor cell growth in a dose-dependent manner in GB cell lines. In addition, it displayed a more effective anti-tumor activity than current standard drug cisplatin in both GB cell lines U87 and LN229. Gene expression profiling revealed that this effect is due to the suppression of multiple kinase signaling pathways regulating *VEGF* levels in GB cells suggesting the possible anti-angiogenic effect. Additionally, inhibition of *HMOX1* abrogates VEGF-induced endothelial activation and subsequent angiogenesis [33]. A robust silencing of *HMOX1* observed in our study convinces curbing of VEGF-induced pathogenic angiogenesis.

Virtual binding studies using bioinformatics tools suggests *EGFR* as a key target of **5a**, exerting its effect through multiple pathways leading to the inhibition of angiogenesis, cell cycle arrest, and apoptosis. Considering previous reports that *EGFR* can activate β-catenin via receptor tyrosine kinase-PI3K/Akt pathway [34], inhibitory ligand binding on *EGFR* is expected to diminish WNT signaling. Our observation of weakened expression of *CTNNB1* and other positive regulators of WNT pathway (*DAB2*, *GSKIP*) corresponds to this notion.

The candidate drug **5a** seems to be an attractive anti-angiogenic agent acting beyond VEGF/VEGFR pathway. Notable downregulation of growth factors (*VEGFD*, *FGF2*, *PGF*, *TNF-α*) and *cytokine IL-1A* was observed in tumor cells upon treatment with 5a. FGF can act synergistically with *VEGF* to amplify tumor angiogenesis and are implicated in the emerging phenomenon of resistance to VEGF inhibition. FGFs have been reported to have potent proangiogenic effects through the stimulation and release of other proangiogenic factors [35]. **5a** was found to effectively reduce *FGF* levels, causing a synergistic anti-angiogenic effect as well as increased sensitization to *VEGF* suppression. Additionally, potent inhibition of *TNF-α* by **5a** may also contribute to its anti-angiogenic impact. Though *TNF* is widely promoted as a antineoplastic agent, its dual role as a angiogenesis promoter and inhibitor has been discussed in multiple reports [36,37,38]. A study by Giraudo et al. discussed the *VEGF* mediated role of *TNF-α* in the initiation and maintenance of angiogenesis and increased vascular permeability [39]. Furthermore, the investigative drug also effectively induced chemo-sensitization by suppressing stress proteins such as *HSPs, CLU, SNAI1, PTGS2,* and *MCL1*. Taken together, we postulate **5a** as a multi-targeted agent against GB signaling pathway and highlighting its significance as anti-tumor agent. Additionally, **5a** exerts its cytostatic effect via key genes involved in regulation of cell cycle pathways as observed in our experimental results. The present research thus suggests **5a** as a candidate GB chemotherapeutic with multiple anti-cancer properties.

## 5. Conclusions

Drugs that can act as multi-targeted agents can enhance efficacy and confront chemoresistance exhibited by GB cells. Thioesters has been investigated as an antitumor agent in multiple studies [40,41]. The present study validates potential of a novel orthothioester **5a**, as an excellent pharmacological scaffold possessing strong cytotoxic, anti-angiogenic, and chemo-sensitization activity. The compound **5a** exerted extensive killing effects on two different glioma cell lines by effectively weakening resistance pathways and enhancing apoptotic machinery. Additionally, **5a** is a strong cytostatic agent acting on key genes involved in regulation of cell cycle pathways. In particular, the capability of **5a** to impede various pathogenic signaling cascades leading to GB pathogenesis, by acting on multiple tyrosine kinase pathways, makes it an appealing anti-GB agent. Taken together, our report provides new insights on how underexplored thioester derivatives can act as a potent pharmacological scaffold against glioblastoma.

## Figures and Tables

**Table 1:** Key genes affected by the top drug-like compound.

Key Genes Dysregulated in Major Pathways						
Signalling Pathways	MAPK	TNF	PI3K-Akt	STAT	ErbB	NF-kB
	AREG	CASP3	AREG	CCND3	AREG	BCL10
	CASP3	FOS	CCND3	CDKN1A	CDKN1A	IL1R1
	CRK	IL15	CDKN1A	IL15	CRK	PTGS2
**Downregulated genes**	FGF2	IL18R1	FGF2	MCL1	HBEGF	RIPK1
	FOS	JUN	MCL1	STAT1	JUN	TNF
	IL1A	JUNB	PGF			
	IL1R1	PTGS2	SGK1			
	JUN	RIPK1	THBS1			
	LAMTOR3	TNF				
	PGF					
	TNF					
	DUSP6	JAG1	BCL2L1	BCL2L1	EGFR	BCL2L1
	EGFR	LIF	EGFR	EGFR	MYC	
	EPHA2	RPS6KA5	EPHA2	LIF	TGFA	
	MET		F2R	MYC		
	MYC		GNG4	PDGFA		
	PDGFA		IRS1	PDGFB		
Upregulated genes	PDGFB		ITGA6	PTPN2		
	RPS6KA5		MET			
	STMN1		MYC			
	TGFA		PDGFA			
	VEGFA		PDGFB			
	BMP4		TGFA			
	RASA3		VEGFA			

## Supplementary Materials

The following are available online at https://www.mdpi.com/2073-4409/8/12/1624/s1: NMR spectroscopy results of previously unreported compounds are attached as Supplementary file 1. List of differentially expressed genes and pathway analysis are attached as Supplementary file 2.

## Abbreviations

GB	Glioblastoma
TKI	Tyrosine Kinase Inhibitor
RTK	Receptor Tyrosine Kinases
ROS	Reactive Oxygen Species
GO	Gene Ontology
TLC	Thin-Layer Chromatography
DCM	Dichloromethane
TCF	Tetrahydrofuran
DE	Differential expression
DEG	Differentially expressed gene
