# Erratum: Viswanathan, A., et al. Battling Glioblastoma: A Novel Tyrosine Kinase Inhibitor with Multi-Dimensional Anti-Tumor Effect (Running Title: Cancer Cells Death Signalling Activation). *Cells* 2019, *8*, 1624

**DOI:** 10.3390/cells9122631

**Published:** 2020-12-07

**Authors:** Anisha Viswanathan, Aliyu Musa, Akshaya Murugesan, João R. Vale, Carlos A. M. Afonso, Saravanan Konda Mani, Olli Yli-Harja, Nuno R. Candeias, Meenakshisundaram Kandhavelu

**Affiliations:** 1 Molecular Signaling Lab, Faculty of Medicine and Health Technology, Tampere University and BioMeditech, P.O. Box 553, 33101 Tampere, Finland; anisha.viswanathan@tuni.fi (A.V.); akshaya.murugesan@tuni.fi (A.M.); 2 Tays Cancer Center, Tampere University Hospital, 33520 Tampere, Finland; 3 Predictive Medicine and Data Analytics Lab, Faculty of Medicine and Health Technology, Tampere University and BioMediTech, P.O. Box 553, 33101 Tampere, Finland; aliyu.musa@tuni.fi; 4 Department of Biotechnology, Lady Doak College, Madurai 625002, India; 5 Faculty of Engineering and Natural Sciences, Tampere University, 33101 Tampere, Finland; rafael.camposdovale@tuni.fi; 6 Instituto de Investigação do Medicamento (iMed.ULisboa), Faculdade de Farmácia, Universidade de Lisboa, Av. Prof. Gama Pinto, 1649-003 Lisboa, Portugal; carlosafonso@ff.ulisboa.pt; 7 Shenzhen Institutes of Advanced Technology, Chinese Academy of Sciences, Shenzhen 518055, China; saravananbioinform@gmail.com; 8 Computational Systems Biology Group, Faculty of Medicine and Health Technology, Tampere University and BioMediTech, P.O. Box 553, 33101 Tampere, Finland; olli.yli-harja@tuni.fi; 9 Institute for Systems Biology, 1441N 34th Street, Seattle, WA 98103-8904, USA

There is an error in the title of the paper [1]. The title of the article should be changed from “Battling Glioblastoma: A Novel Tyrosine Kinase Inhibitor with Multi-Dimensional Anti-Tumor Effect (Running Title: Cancer Cells Death Signalling Activation)” to “Battling Glioblastoma: A Novel Tyrosine Kinase Inhibitor with Multi-Dimensional Anti-Tumor Effect”. 

In addition, the first affiliation “Molecular Signaling Lab, Faculty of Medicine and Health Technology, Tampere University, BioMeditech and Tays Cancer Center, Tampere University Hospital, P.O. Box 553, 33101 Tampere, Finland” should be split into two:

^1^  Molecular Signaling Lab, Faculty of Medicine and Health Technology, Tampere University, BioMeditech and Tays Cancer Center, Tampere University Hospital, P.O. Box 553, 33101 Tampere, Finland
^2^  Predictive Medicine and Data Analytics Lab, Faculty of Medicine and Health Technology, Tampere University and BioMediTech, P.O. Box 553, 33101 Tampere, Finland

Thus, the original affiliation orders 2–8 should be changed to 3–9, respectively. The correct version is listed below:

Anisha Viswanathan ^1,2^, Aliyu Musa ^3^, Akshaya Murugesan ^1,2,4^, João R. Vale ^5,6^, Carlos A. M. Afonso ^6^, Saravanan Konda Mani ^7^, Olli Yli-Harja ^8,9^, Nuno R. Candeias ^5,^* and Meenakshisundaram Kandhavelu ^1,2,^*

^1^  Molecular Signaling Lab, Faculty of Medicine and Health Technology, Tampere University and BioMeditech, P.O. Box 553, 33101 Tampere, Finland
^2^  Tays Cancer Center, Tampere University Hospital, 33520 Tampere, Finland
^3^  Predictive Medicine and Data Analytics Lab, Faculty of Medicine and Health Technology, Tampere University and BioMediTech, P.O. Box 553, 33101 Tampere, Finland
^4^  Department of Biotechnology, Lady Doak College, Madurai 625002, India
^5^  Faculty of Engineering and Natural Sciences, Tampere University, 33101 Tampere, Finland
^6^  Instituto de Investigação do Medicamento (iMed.ULisboa), Faculdade de Farmácia, Universidade de Lisboa, Av. Prof. Gama Pinto, 1649-003 Lisboa, Portugal
^7^  Shenzhen Institutes of Advanced Technology, Chinese Academy of Sciences, Shenzhen 518055, China
^8^  Computational Systems Biology Group, Faculty of Medicine and Health Technology, Tampere University and BioMediTech, P.O. Box 553, 33101 Tampere, Finland
^9^  Institute for Systems Biology, 1441N 34th Street, Seattle, WA 98103-8904, USA

We apologize for this error and state that the scientific conclusions are unaffected. The original article has been updated.
