# Supplementary material for: Battling Glioblastoma: A Novel Tyrosine Kinase Inhibitor with Multi-Dimensional Anti-Tumor Effect (Running Title: Cancer Cells Death Signalling Activation)
Source: Cells. 2019 Dec 12;8(12):1624. doi: 10.3390/cells8121624 (PMC6953096; doi:10.3390/cells8121624)
Supplement: Supplementary file 1 [file cells-08-01624-s001.zip › Supplementary file 1.docx]

Battling Glioblastoma: A Novel Tyrosine Kinase Inhibitor with Multi-dimensional Anti-tumor Effect

Anisha Viswanathan^1^, Aliyu Musa^2^, Akshaya Murugesan^1,3^, João R. Vale^4,5^, Carlos A. M. Afonso^5^, Saravanan Konda Mani^6^, Olli Yli-Harja^7,8^, Nuno R. Candeias^4^* and Meenakshisundaram Kandhavelu^1,^*

^1^ Molecular Signaling Lab, Faculty of Medicine and Health Technology, Tampere University, BioMeditech and Tays Cancer Center, Tampere University Hospital, P.O. Box 553, 33101 Tampere, Finland.

^2^ Predictive Medicine and Data Analytics Lab, Faculty of Medicine and Health Technology, Tampere University and BioMediTech, P.O. Box 553, 33101 Tampere, Finland.

^3^ Department of Biotechnology, Lady Doak College, Thallakulam, Madurai – 625002, India.

^4^ Faculty of Engineering and Natural Sciences, Tampere University, 33101 Tampere, Finland

^5^ Instituto de Investigação do Medicamento (iMed.ULisboa), Faculdade de Farmácia, Universidade de Lisboa, Av. Prof. Gama Pinto, 1649-003 Lisboa, Portugal

^6^ Shenzhen Institutes of Advanced Technology, Chinese Academy of Sciences, Shenzhen, Guangdong, China

^7^ Computational Systems Biology Group, Faculty of Medicine and Health Technology, Tampere University and BioMediTech, P.O. Box 553, 33101 Tampere, Finland.

^8^ Institute for Systems Biology, 1441N 34^th^ Street, Seattle, WA 98103-8904, USA

***** Correspondence: [meenakshisundaram.kandhavelu@tuni.fi](mailto:meenakshisundaram.kandhavelu@tuni.fi); Tel.: (+358)417488772

**NMR spectra of unreported compounds**

**5a,** ^1^H NMR (500 MHz, CDCl_3_)

**5a,** ^13^C NMR (125 MHz, CDCl_3_)

**5b,** ^1^H NMR (500 MHz, CDCl_3_)

**5b,** ^13^C NMR (125 MHz, CDCl_3_)
